# Diabetes in Humans Activates Pancreatic Stellate Cells via RAGE in Pancreatic Ductal Adenocarcinoma

**DOI:** 10.3390/ijms222111716

**Published:** 2021-10-28

**Authors:** Chiaki Uchida, Hiroki Mizukami, Yutaro Hara, Takeshi Saito, Satoko Umetsu, Akiko Igawa, Sho Osonoi, Kazuhiro Kudoh, Yasuhiko Yamamoto, Hiroshi Yamamoto, Soroku Yagihashi, Kenichi Hakamada

**Affiliations:** 1 Department of Pathology and Molecular Medicine, Hirosaki University Graduate School of Medicine, Hirosaki 036-8562, Japan; doumobokudesu_gananika@yahoo.co.jp (C.U.); y.hara@hirosaki-u.ac.jp (Y.H.); igawa-a@hirosaki-u.ac.jp (A.I.); s.osonoi@hirosaki-u.ac.jp (S.O.); spkk-spkk@umin.ac.jp (K.K.); yagihasi@hirosaki-u.ac.jp (S.Y.); 2 Department of Gasrtroenterological and Pediatric Surgery, Hirosaki University Graduate School of Medicine, Hirosaki 036-8562, Japan; t-saito_0622_@kjb.biglobe.ne.jp (T.S.); satomakotoko@gmail.com (S.U.); hakamada@hirosaki-u.ac.jp (K.H.); 3 Department of Biochemistry and Molecular Vascular Biology, Kanazawa University Graduate School of Medical Sciences, Kanazawa 920-8640, Japan; yasuyama@med.kanazawa-u.ac.jp (Y.Y.); hiroshi.yamamoto@komatsu-u.ac.jp (H.Y.); 4 Komatsu University, Komatsu 923-0921, Japan

**Keywords:** type 2 diabetes, advanced glycation end products, pancreatic ductal adenocarcinoma, RAGE, EMT, metabolic syndrome

## Abstract

Pancreatic stellate cells (PSCs) mainly consist of cancer-associating fibroblasts in pancreatic ductal adenocarcinoma (PDAC). The receptor for advanced glycation end products (RAGE) is implicated in the pathophysiology of diabetic complications. Here, we studied the implication of RAGE in PSC activation in PDAC. The activation of cultured mouse PSCs was evaluated by qPCR. The induction of epithelial mesenchymal transition (EMT) in PDAC cell lines was assessed under stimulation with culture supernatant from activated PSCs. A total of 155 surgically resected PDAC subjects (83 nondiabetic, 18 with ≦3-years and 54 with >3-years history of diabetes) were clinicopathologically evaluated. A high-fat diet increased the expression of activated markers in cultured PSCs, which was abrogated by RAGE deletion. Culture supernatant from activated PSCs facilitated EMT of PDAC cells with elevation of TGF−β and IL−6, but not from RAGE−deleted PSCs. Diabetic subjects complicated with metabolic syndrome, divided by cluster analysis, showed higher PSC activation and RAGE expression. In such groups, PDAC cells exhibited an EMT nature. The complication of metabolic syndrome with diabetes significantly worsened disease−free survival of PDAC subjects. Thus, RAGE in PSCs can be viewed as a new promoter and a future therapeutic target of PDAC in diabetic subjects with metabolic syndrome.

## 1. Introduction

Pancreatic ductal adenocarcinoma (PDAC), one of the most difficult diseases in medicine, remains the fourth leading cause of cancer-related death worldwide [1,2]. Type 2 diabetes (T2D) is known to be a risk factor for the development of PDAC in both Japanese and Caucasians [3,4]. Epigenetic modifications are candidates for explaining the close relationship between diabetes and PDAC [5], while the detailed mechanisms are still not fully elucidated. 

The stroma in PDAC, which can account for as much as 80–90% of the tumour mass, is partially associated with the proliferation of pancreatic stellate cells (PSCs). In the pancreas, PSCs are the main progenitors of cancer-associated fibroblasts (CAFs) [6,7,8]. PSCs transform from quiescent cells to activated myofibroblast-like cells (MFCs) through various stimuli, including hyperglycaemia, obesity, and hyperinsulinaemia, in which secreted cytokines from activated PSCs promote tumour cell proliferation and invasion, metastasis, epithelial−mesenchymal transition (EMT) and resistance to chemotherapy [9,10,11,12,13,14,15,16,17,18,19]. In T2D models, abnormal pancreatic fibrosis was also observed with the proliferation of alpha smooth muscle actin (αSMA)−positive cells [20,21]. Although T2D often manifests as a part of metabolic syndrome, including obesity and dyslipidaemia, it is still unknown whether these metabolic disturbances are synergistically or additionally involved in the activation of PSCs in PDAC.

The receptor for advanced glycation end products (RAGE) is a multiligand receptor implicated in inflammation and proliferation and is one of the main receptors for advanced glycation end products (AGEs) [22]. Numerous studies have reported a direct correlation between the AGE-RAGE axis and the development and progression of diabetic vascular complications [23]. In rat hepatic stellate cells (HSCs), the expression of RAGE is upregulated with the transition to myofibroblast-like cells (MFBs), which suggests that RAGE can play a major role in hepatic fibrosis [24]. PSC activation via RAGE relates to tumour growth in PDAC cells [25]. Nevertheless, the role of RAGE in PSCs is not fully elucidated in PDAC complicated with T2D and metabolic syndrome. Herein, we evaluated the role of RAGEs on PSCs in PDAC complicated with T2D and metabolic syndrome in this study.

## 2. Results

### 2.1. Effects of High Fat Diet on the Activation of PSCs Isolated from Experimental Mice

**Figure 1:**
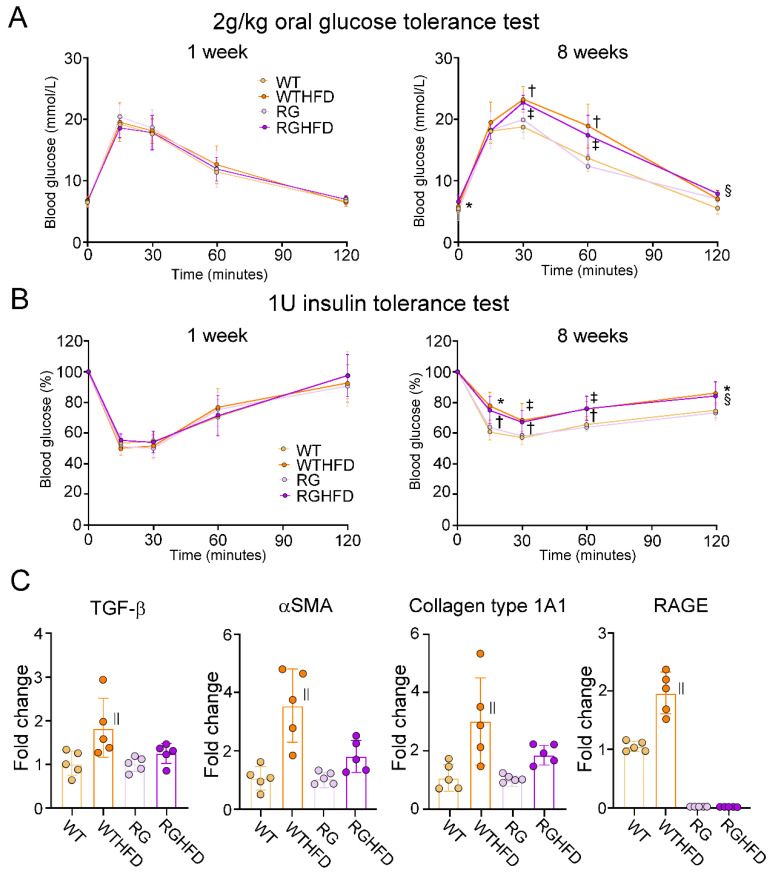
Effects of HFD on activation of PSCs isolated from WT and RG. Oral glucose tolerance test revealed that glucose tolerance was similar among all groups at the beginning of each week 1 (**A**). Eight weeks after HFD feeding, glucose tolerance was significantly aggravated in both WT and RG compared to those fed a normal diet. An insulin tolerance test revealed that insulin tolerance was comparable among all groups at the starting points (**B**). HFD feeding significantly aggravated insulin tolerance compared to normal diet feeding in both WT and RG. HFD feeding significantly increased the mRNA expression of TGF−β, αSMA, Collagen 1A1 and RAGE in PSCs isolated from WT mice (**C**). In contrast, HFD feeding had no significant impacts on the expression of those genes in PSCs isolated from RG. RAGE expression was almost null in RG. Values are presented as means ± SD. WT: Wild type, WTHFD: High fat diet−fed WT, RG: RAGE−null mice, RGHFD: High fat diet−fed RG. * *p* < 0.05 RG compared with RGHFD, ^†^ *p* < 0.01 WT compared with WTHFD, ^‡^ *p* < 0.01 RG compared with RGHFD, ^§^ *p* < 0.05 WT compared with WTHFD, ^||^ *p* < 0.05 compared with WT.

First, we evaluated the effects of abnormal glucose tolerance and metabolic syndrome on PSC activation in high fat diet (HFD)−induced obese (DIO) mice. The body weight of the experimental animals was comparable among all groups at the starting point (Table 1). After eight weeks, the body weight of the HFD−fed wild type littermate mice (WTHFD) was significantly higher than that of wild type littermate mice (WT) fed with normal diet (ND). The body weight of the HFD−fed RAGE-null mice (RGHFD) was similarly higher than that of ND−fed RAGE-null mice (RG). Fasting and nonfasting blood glucose also increased in both of the HFD-fed groups compared to the ND−fed groups. Both triglyceride (TG) and total cholesterol (TC) were almost 2 times higher in the WTHFD RGHFD compared to the ND group. HFD feeding similarly increased fasting plasma insulin levels in the WT and RG groups compared to the ND groups. Glucose tolerance was comparable among all groups at the starting point (Figure 1A). In contrast, at eight weeks, HFD feeding significantly aggravated glucose tolerance in WT and RG compared to ND feeding. Insulin tolerance was comparable among all groups at the starting points (Figure 1B). HFD feedings significantly attenuated insulin tolerance compared to ND feeding in WT and RG. To evaluate the effects of HFD feedings on PSC activation, PSCs were isolated from the pancreata of experimental animals at the end of high-fat feedings. There was spindle-cell morphology of isolated PSCs (Supplementary Figure S1A). These cells contained lipid droplets in their cytoplasm, as shown by oil red O staining (Supplementary Figure S1B). HFD feeding significantly increased the mRNA expression of TGF−β, αSMA, collagen type 1A1 and RAGE in the WT group (Figure 1C). In contrast, the RG group showed no significant increase in the expression of those genes with HFD feeding. RAGE expression was almost null in RG and RGHFD PSCs. These results suggested that PSCs were activated by HFD via RAGE.

### 2.2. AGE Stimulation Activated PSCs via RAGE

**Figure 2:**
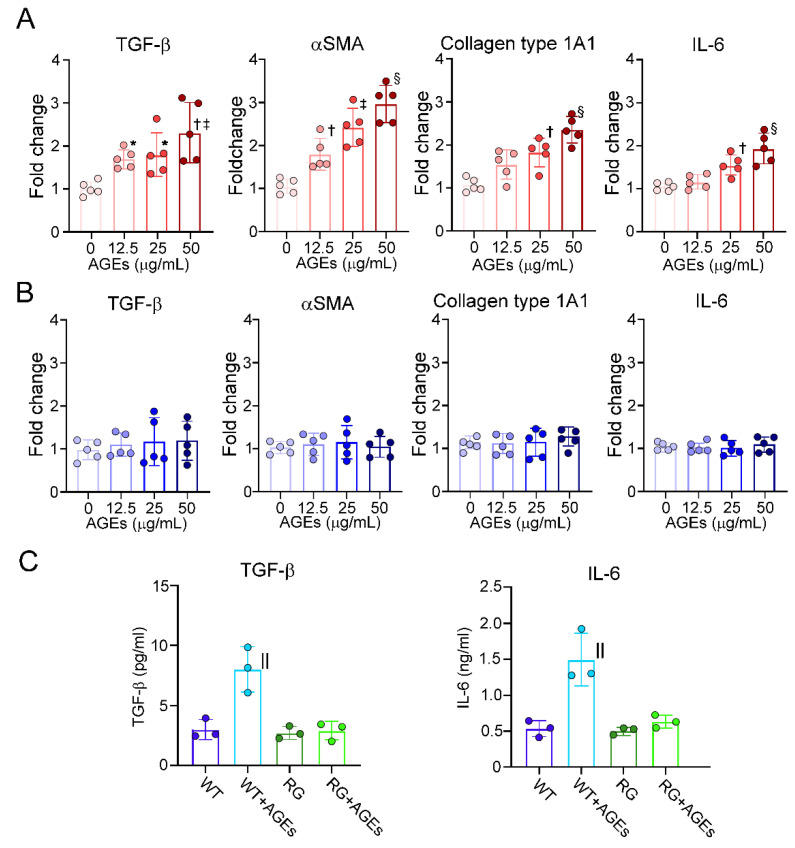
Humoral factors derived from PSCs stimulated by AGEs. AGEs stimulation increased the mRNA expression of TGF−β, αSMA, collagen type 1A1 and IL−6 in a dose-dependent manner in PSCs isolated from WT mice (**A**). AGEs had no effects on the mRNA expression of those genes in PSCs isolated from RG (**B**). AGEs stimulation increased the concentrations of TGF−β and IL−6 in the supernatant of WT PSC culture medium, while the concentration was not changed in the supernatant of RG PSC culture medium (**C**). Values are presented as means ± SD. AGEs: Advanced glycation end products, WT: PSCs isolated from wild−type mice, RG: PSCs isolated from RAGE−deleted mice. * *p* < 0.05 compared with 0 μg/mL, ^†^ *p* < 0.01 compared with 0 μg/mL, ^‡^ *p* < 0.05 compared with 12.5 μg/mL, ^§^ *p* < 0.05 compared with 25 μg/mL, ^||^ *p* < 0.01 vs. WT.

To evaluate the direct effects of AGEs on the activation of PSCs, PSCs isolated from WT and RG were stimulated with various doses of AGEs. AGE stimulation increased the mRNA expression of TGF−β, αSMA, collagen type 1A1 and IL−6 in WT PSCs in a dose-dependent manner, between 12.5 µg/mL and 50 µg/mL in PSCs isolated from WT PSCs (Figure 2A). In contrast, the effects of AGEs were eradicated on PSCs isolated from RG (Figure 2B). The concentrations of TGF−β and IL−6 were significantly increased in the supernatant of PSCs isolated from WT stimulated with 50 µg/mL AGEs (Figure 2C). On the other hand, AGEs stimulation had no impacts on the concentration in the supernatant of RG.

### 2.3. Humoral Factors Derived from PSCs Stimulated with AGEs Evoked EMT in PDAC

**Figure 3:**
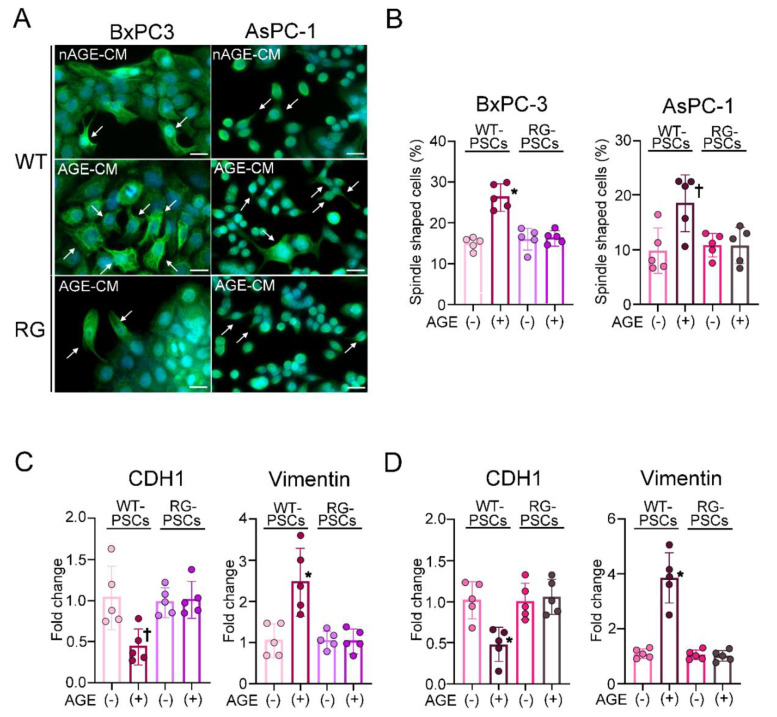
Evaluation of EMT in PDAC cells elicited by coculture with activated PSCs. AGE−CM could transform the cell contour of BxPC−3 cells and the AsPC−1 cell line into a spindle shape, as depicted by immunofluorescence for cytokeratin AE1/AE3, compared with BxPC−3 and AsPC−1 cells cultured with nAGE−CM (**A**). AGE-CM derived from RG PSCs could not transform the cell contours of both kinds of PDAC cells. The frequency of the transformed cells was significantly increased in AGE−CM compared with nAGE−CM in both BxPC−3 and AsPC−1 cells, while the frequency was comparable between nAGE−CM and AGE−CM derived from RG−PSCs (**B**). The mRNA expression of CDH−1 was significantly reduced and the expression of vimentin was significantly increased in BxPC−3 (**C**) and AsPC−1 (**D**) cells cultured with AGE−CM compared to nAGE−CM. RAGE deletion in PSCs abolished the effects of AGE−CM on the expression of CDH−1 and vimentin in BxPC−3 (**C**) and AsPC−1 (**D**) cells. Values are presented as means ± SD. WT: Wild type mice, RG: RAGE−null mice, PSCs: Pancreatic stellate cells, AGEs: Advanced glycation end products, nAGE−CMA: Medium cultured with nonstimulated PSCs, AGE−CM: Medium cultured with AGE−stimulated PSCs, PDAC: Pancreatic ductal adenocarcinoma. * *p* < 0.01 compared with nAGE−CM, ^†^ *p* < 0.05 compared with nAGE−CM. The scale bar represents 25 μm.

The human PDAC cell lines BxPC−3 and AsPC−1, cultured with conditioned media derived from the culture supernatant of AGE−stimulated PSCs (AGE−CM) or non-stimulated PSCs (nAGE−CM) for 24 h, transformed into spindle shapes similar to mesenchymal cells, as depicted by immunofluorescence for cytokeratin AE1/AE3 (Figure 3A). The frequency of transformed cells was 1.7 times and 1.9 times increased in AGE−CM compared to nAGE−CM (Figure 3B). The mRNA expression of CDH−1 was reduced by almost half and that of vimentin was 2.5 times elevated in BxPC−3 cells cultured with AGE−CM compared to nAGE−CM (Figure 3C). Coculture with AGE−CM similarly decreased the mRNA expression of CDH−1 and 3.9 times increased that of vimentin in AsPC−1 cells compared to coculture with nAGE−CM (Figure 3D). The induction of EMT was cancelled if BxPC−3 and AsPC−1 cells were cultured with AGE−CM derived from the culture supernatant of RG PSCs (Figure 3A–D).

### 2.4. Increase in αSMA Expression Surrounding Pancreatic Intraepithelial Neoplasia in T2D Patients with Metabolic Syndrome

**Figure 4:**
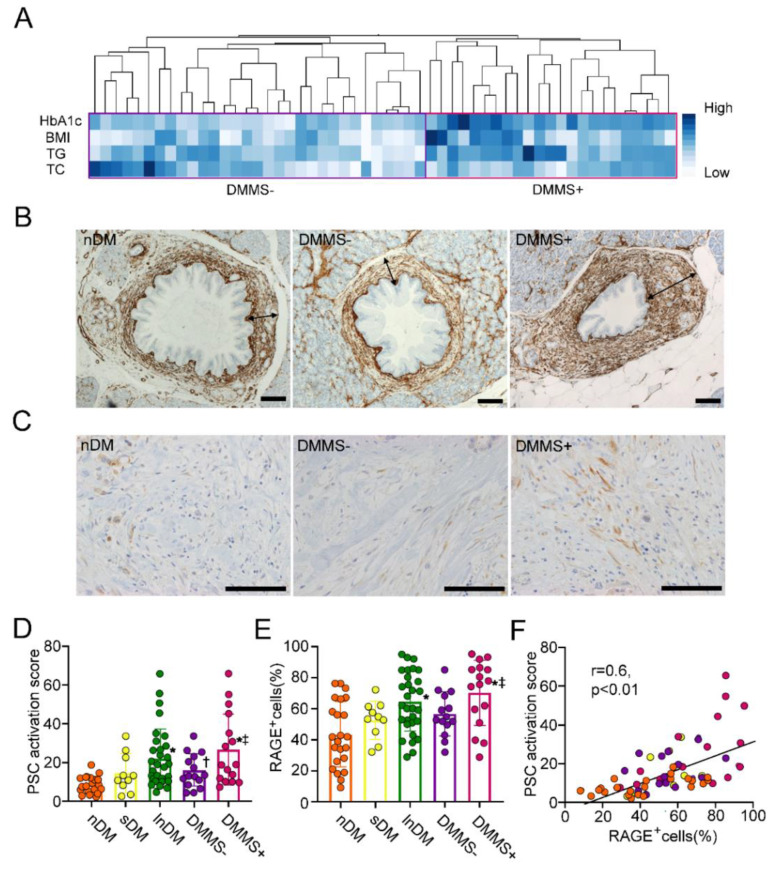
αSMA expression surrounding PanIN in T2D patients with metabolic syndrome. The hierarchical cluster analysis on the signature of HbA1c, BMI, TG, and TC yielded two distinct groups (DMMS− and DMMS+) (**A**). The PSC activation score was comparable between nDM and sDM subjects, but it marginally increased in DMMS− compared with nDM subjects (*p* < 0.049). (**B**,**D**). lnDM and DMMS+ showed a further increase in the expression of αSMA surrounding PanIN compared with nDM. DMMS+ showed a significant increase in the expression of αSMA surrounding PanIN compared with DMMS−. RAGE was expressed in PDAC cells and stromal cells (**C**). The frequency of RAGE−positive stromal cells was comparable between nDM and sDM but marginally increased in DMMS− compared to nDM (**C**,**E**). Those were further increased in DMMS+ and lnDM compared with nDM. DMMS+ showed significantly higher prevalence than DMMS−. The frequency of RAGE−positive stromal cells was proportionally correlated with the PSC activation score (**F**). Values are presented as means ± SD. TG: Triglyceride, TC: Total cholesterol, nDM, Nondiabetic subjects, lnDM: Long-term type 2 diabetic subjects, DMMS−: lnDM without metabolic syndrome, DMMS+: lnDM complicated with metabolic syndrome, RAGE: receptor for advanced glycation end product. * *p* < 0.01 compared with nDM, ^†^ *p* < 0.05 compared with nDM and ^‡^ *p* < 0.05 compared with DMMS−. Arrows show the maximum width of the αSMA−positive area. The scale bar represents 40 μm (**B**) and 10 μm (**C**).

Surgically resected PDAC subjects divided into lnDM (more than 3-year duration of T2D), sDM (3 years or less duration of T2D) and non−T2D complicated PDAC subjects (nDM) were examined (Table 2). Average age, sex, and BMI were comparable between the nDM and lnDM groups. HbA1c was significantly increased in lnDM compared to nDM. lnDM showed a higher prevalence of worsened histological grade (poorly differentiated adenocarcinoma:por) and T stage than nDM (*p* < 0.01 and *p* < 0.05, respectively). N factors based on UICC, 8th edition, and classification were comparable between nDM and lnDM. sDM did not worsen any clinicopathological measures compared to nDM except for HbA1c. sDM showed a lower prevalence of worsened histological grade (por) and T stage than lnDM (*p* < 0.01 and *p* < 0.05, respectively). To examine the implications of the factors related to metabolic syndrome, HbA1c, BMI, triglyceride (TG), and total cholesterol (TC) in the activation of PSCs, hierarchical cluster analysis on the signature of these factors was conducted to see if there was a relationship to fibrosis in PDAC. There was a profile that yielded two distinct groups (DMMS− and DMMS+) (Figure 4A). The detailed clinical profile of DMMS+ patients is shown in Table 3. DMMS+ displayed high scores for all factors excluding HbA1c. Histologic grade and T stage were significantly worse in DMMS+ patients than in DMMS− patients (*p* < 0.01 and *p* < 0.05, respectively). When the mean value of αSMA expression surrounding pancreatic intraepithelial neoplasia (PanIN) as a PSC activation score was compared among the groups, the score was comparable between nDM and sDM (Figure 4B,D). There was a significant increase in the score in lnDM, DMMS− and DMMS+ patients compared with nDM patients (Figure 4B,D). DMMS+ showed a significant increase in the expression of αSMA surrounding PanIN compared with DMMS− (*p* < 0.05). The frequency of RAGE−positive stromal cells evaluated in the RAGE−immunostained sections was comparable in nDM and sDM (Figure 4C,E). The frequency mildly, but not significantly increased in DMMS− compared to nDM (Figure 4C,E). Those were further increased in DMMS+ and lnDM compared with in nDM. The frequency of DMMS+ was significantly increased compared to that of DMMS− (*p* < 0.05). The frequency of RAGE−positive stromal cells was significantly correlated with the degrees of αSMA expression surrounding PanIN (Figure 4F). These results could suggest that T2D with a duration of more than three years but not a short duration of diabetes had significant impacts on stromal activation concurrently with a high prevalence of RAGE−expressing stromal cells.

### 2.5. Expression of EMT Markers in PDAC Cells

**Figure 5:**
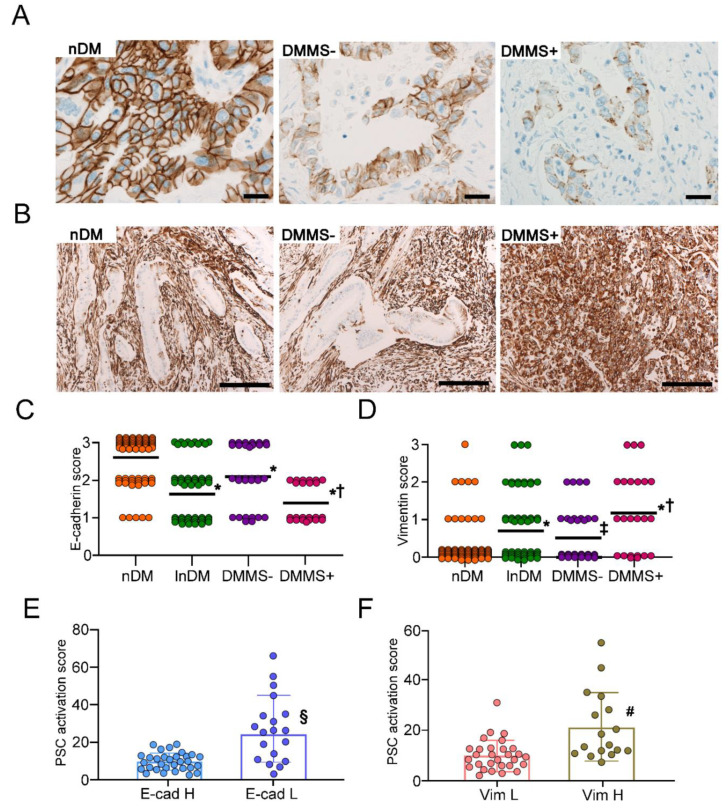
Expression of EMT markers in PDAC cells. The expression of E−cadherin was reduced in lnDM and DMMS– compared to nDM (**A**,**C**). The expression was further decreased in DMMS+ compared to DMMS−. The expression of vimentin in PDAC cells was significantly increased in lnDM and DMMS– compared to nDM (**B**,**D**). DMMS+ showed higher expression of vimentin than DMMS−. The PSC activation score was significantly increased in E−cad L compared to E−cad H (**E**). The PSC activation score was significantly increased in Vim H compared to Vim L (**F**). Values are presented as means ± SD. nDM: nondiabetic subjects, lnDM: Long−term type 2 diabetic subjects, DMMS−: lnDM without metabolic syndrome, DMMS+: lnDM complicated with metabolic syndrome, E−cad H: E−cadherin highly expressed subjects, E−cad L: E−cadherin low expressed subjects, Vim L: Vimentin low expressed subjects, Vim H: Vimentin highly expressed subjects. * *p* < 0.01 compared with nDM, ^†^ *p* < 0.05 compared with DMMS–, ^‡^ *p* < 0.05 compared with nDM, ^§^ *p* < 0.01 compared with E−cad H, ^#^ *p* < 0.01 compared with Vim L. The scale bar represents 10 μm.

Immunohistochemical staining revealed the expression of E-cadherin on the cell membrane of PDAC cells, nonneoplastic ductal cells, islet cells and acinar cells (Figure 5A). The semiquantitative expression of E−cadherin in PDAC cells revealed that the expression was significantly reduced in lnDM, DMMS– and DMMS+ compared to nDM (*p* < 0.01) (Figure 5A,C). E−cadherin expression in PDAC cells of DMMS+ was significantly decreased compared to DMMS− (*p* < 0.05). In contrast, vimentin expression was mainly observed in the cytosol of stromal cells (Figure 5B). Vimentin expression in PDAC cells was significantly increased in lnDM compared to nDM (*p* < 0.01) (Figure 5B,D). The expression of lnDM was comparable with DMMS−. However, the expression in DMMS+ was significantly increased compared to that in DMMS− (*p* < 0.05). If E−cad H was defined as the cases showing an E−cadherin score of 3, the PSC activation score was significantly increased in E−cad L compared to E−cad H (*p* < 0.01) (Figure 5E). If Vim H was defined as the cases showing vimentin scores of 2 or 3 in PDAC, the PSC activation score was significantly increased in Vim H compared to Vim L (*p* < 0.01) (Figure 5F). 

### 2.6. Diabetes Complicated with Metabolic Syndrome and EMT Are Associated with Short Disease Free Survival or Overall Survival in PDAC

**Figure 6:**
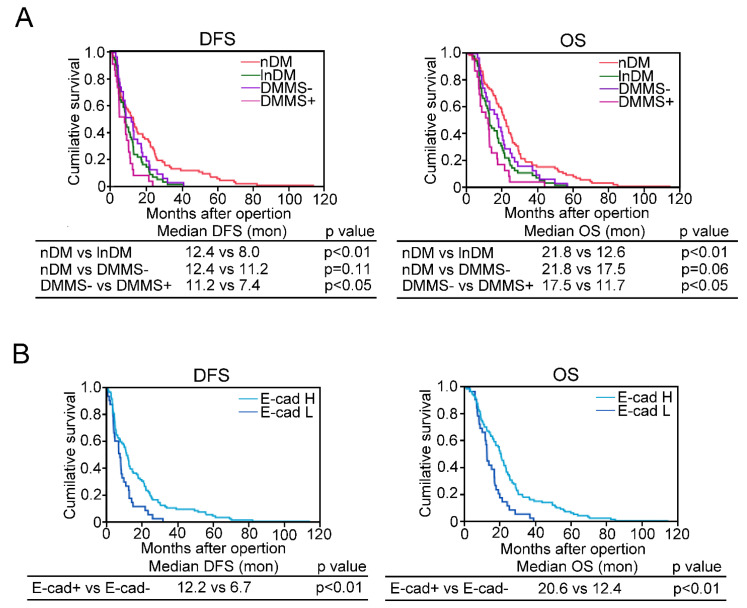
Prognosis of PDAC subjects complicated with T2D and metabolic syndrome. Kaplan−Meier survival curves showed a shortened DFS and OS in lnDM compared to nDM (**A**). OS of DMMS− were marginally shortened compared with those of nDM, while both for DMMS+ were much shorter than those of DMMS−. The reduction of E−cadherin expression in PDAC cells also shortened DFS and OS (**B**). nDM: nondiabetic subjects, lnDM: Long−term type 2 diabetic subjects, DMMS−: lnDM without metabolic syndrome, DMMS+: lnDM complicated with metabolic syndrome, E−cad H: E−cadherin highly expressed subjects, E−cad L: E−cadherin low expressed subjects.

Univariate analysis for disease free survival (DFS) showed that T2D history, high HbA1c (≧7%), DMMS+, low expression of E-cadherin, and high expression of vimentin were the most significant risk factors for DFS (Table 4), while they were also significant risk factors for reduced overall survival (OS) (Table 5). Multivariate analysis further confirmed that low expression of E−cadherin remained a significant risk factor for DFS and OS (Table 6 and Table 7). The Kaplan-Meier survival curve clearly indicated a shortened DFS and OS in lnDM compared to nDM (Figure 6A). In DMMS–, both DFS and OS were marginally shortened compared to nDM, but not with a significant difference, while both in DMMS+ were significantly shorter than in DMMS– (*p* < 0.05, respectively). A reduction in E−cadherin expression in PDAC cells also shortened DFS and OS (*p* < 0.01) (Figure 6B).

## 3. Discussion

The activation and expression of RAGE are implicated in the proliferative, inflammatory, fibrotic reactions with transdifferentiation of cultured HSCs into MFBs [24]. In contrast to HSCs, the role of RAGE in PSCs is not fully understood. Miller-Ocuin J.L. et al. showed that PSCs express RAGE, which binds to extracellular DNA from neutrophils and activates PSCs [25]. We confirmed that AGEs elicited transdifferentiation of PSCs into MFBs in the presence of RAGE. Furthermore, the prevalence of RAGE-expressing mesenchymal cells was significantly correlated with the degree of αSMA expression surrounding PanIN in surgical specimens of PDAC. Thus, activation of RAGE in PSCs can be assumed to trigger MFB transdifferentiation of PSCs in PDAC.

PSCs are activated in obesity and hyperglycaemic states through various mechanisms, such as inflammation, oxidative stress, MAP kinases activation, and insulin signalling [12,13,14,15,16,17,18]. In this study, the expression of MFB markers and RAGE in PSCs isolated from DIO mice increased more than that of PSCs from WT mice. In DIO, the mild T2D model complicated with metabolic syndrome, including obesity and dyslipidaemia, simulated Caucasian obese subjects [26]. It is well documented that obesity is characterized by chronic, low-grade, sterile inflammation and that macrophages are the predominant immune cells in adipose tissue which contribute to systemic inflammation [27,28,29]. In inflammatory circumstances such as diabetes with obesity, plasma high mobility group box chromosomal protein 1 and S100A4, which are ligands of RAGE, are also increased, accompanied by AGEs [30,31,32]. In addition, the expression of RAGE is upregulated in the adipose tissue of obese subjects [33]. Because nondiabetic subjects with increased RAGE expression did not necessarily show apparent activation of PSCs in our study, upregulation of both RAGE expression and ligands of RAGE would be required to activate PSCs. In this study, PSCs isolated from DIO mice significantly differentiated into MFCs despite mild glucose intolerance with an increase in RAGE expression. Thus, it is likely that chronic inflammation exacerbates in metabolic syndrome and that abnormal glucose tolerance could unitedly make a greater contribution to the activation of RAGE signalling in PSCs than hyperglycaemia alone in PDAC. 

Many previous reports have shown that activated PSCs can promote the invasiveness and metastasis of PDAC via EMT induction [19,24,34,35,36,37,38,39]. We showed that supernatant derived from PSCs stimulated by AGEs could induce EMT in the BxPC−3 and AsPC−1 cell lines, while culture supernatant derived from AGE−stimulated PSCs isolated from RG did not emphasize EMT. Our evaluation with human subjects further supported an in vitro study. These results indicated that humoral factors secreted from PSCs activated by AGEs could induce EMT in PDAC cells. Previous studies have reported that TGF−β, IL−6 and galectin−1 are secreted from PSCs and induce EMT in PDAC cells [19,38,39]. In this study, we confirmed that TGF−β and IL−6 were secreted from PSCs stimulated with AGEs. Thus, suppression of these factors might be a therapeutic option to decrease EMT in PDAC cells complicated with diabetes and metabolic syndromes. 

In this study, the question of why long−term diabetes is associated with PSC activation is not answered. Because long−term consequences of T2D increase the incidences of dyslipidaemia and diabetic complications, it can evoke PSC activation. In contrast, the short duration of diabetes manifested in PDAC could be a secondary diabetes triggered by PDAC [40]. Indeed, our previous report showed that resection of the tumour by pancreato-duodenectomy improved glycated haemoglobin values in PDC patients with short−term diabetes, while none of the patients with long-term diabetes showed improvement [5]. Therefore, since the mechanism of diabetes can be totally different between long−term and short-term diabetes in PDAC, those differences might reflect the different impacts on PSCs. 

In PDAC, CAFs have heterogeneous origins, phenotypes, and functions. A crucial challenge is to understand how much of this heterogeneity serves different biological responses to cancer cells. Mizutani et al. found that meflin was a marker for cancer-restraining CAFs, which in turn could yield meflin−low and αSMA−high expression cancer-promoting CAFs in PDAC [41]. Although the definite factors eliciting this transition have not been identified, previous research shows that ageing, hypoxia, and TGF−β signalling decrease meflin expression [42,43]. Ageing is also a risk factor for diabetes. Furthermore, the obesity and vascular complications of diabetes are known to induce tissue hypoxia, which is associated with tissue dysfunction [44,45]. Therefore, the presence of diabetes and metabolic syndrome is assumed to be involved in the downregulation of meflin expression in PSCs and elicits a change in the CAF population in PDAC.

The OS and DFS of DMMS+ patients were significantly worse than those of DMMS– patients. In human subjects, T2D and metabolic syndrome are risk factors for fat deposition in the pancreas [46]. The terrain of pancreatic steatosis can evoke pancreatic inflammatory processes, which are the most important predisposing factors for the development of PDAC [47]. To date, the carcinogenetic mechanism in fatty pancreas has still not been fully elucidated. Although the detailed correlation between fat deposition in the pancreas and PSC activation should be evaluated in the future, inflammatory signalling from local adipocytes might stimulate RAGE signalling in PSCs by eliciting the EMT phenotype of PDAC cells. Furthermore, our results suggest that comprehensive treatment of T2D and metabolic syndrome would be critical to prevent terrain inflammation and for the better prognosis of PDAC.

Our results have suggested that T2D complicated with metabolic syndrome, such as dyslipidaemia and obesity, could induce EMT with a reduction in the expression of E−cadherin and an increase in the expression of vimentin through RAGE activation in PSCs. Nonetheless, in multivariate analysis, DMMS+ was not a risk factor, while low expression of E−cadherin was a significant risk factor for OS and DFS in PDAC. This finding possibly reflects the idea that factors other than DMMS+ contribute to the decrease in the expression of E−cadherin in PDAC. E−cadherin expression in PDAC is known to be regulated in various different ways, such as transcriptional repression, epigenetic silencing (promoter methylation and micro RNA), gene mutation, endocytosis and also proteolytic cleavage [48]. We previously revealed that a long history of T2D is associated with a high prevalence of promoter methylation of CDH1, resulting in poor prognosis [5]. Therefore, factors other than EMT signalling could be involved in the reduction in the expression of E−cadherin, and worsen the prognosis in PDAC even if PDAC subjects are not complicated with T2D.

There are several limitations in this study. Although the total sample number of PDAC complicated with diabetes was 54 cases, it was not sufficient for multivariate analysis because of the division of the groups based on cluster analysis. For multivariate analysis, higher sample numbers are required. Another drawback of this study could be that the prevalence of PDAC complicated with diabetes and metabolic syndrome was not directly evaluated because of the retrospective nature of the data. For in vitro study, primary human PDAC cells and PSC cells should be used to evaluate the more direct implication of RAGE and diabetes in PSC activation and EMT in PDAC. 

Taken together, our study has provided important information on the close association of long−term diabetes with metabolic syndrome and PSC activation via RAGE with unfavourable prognosis. It is hoped that future studies will explore the possibility of developing a combination therapy in which the correction of metabolic disturbance treatment, promoter methylation recovery and chemotherapy are considered together for PDAC.

## 4. Materials and Methods

### 4.1. Ethical Statement

All investigations and experiments involving human subjects were performed with the permission of the ethical committee of Hirosaki University Graduate School of Medicine (approval number, #2016−0084) and were performed according to the guidelines of the Ethics Committee on human research samples from the Japanese Society of Pathology and the provisions of the Declaration of Helsinki. Informed consent was obtained from all participants and/or their legal guardians.

### 4.2. Animal Study

C57BL/6 RG mice were generated and kindly gifted by Prof. Yamamoto, Kanazawa University Graduate School of Medicine [49]. All mice were maintained under specific pathogen-free conditions and were used for the study upon approval by the Animal Care and Use Committee of the Hirosaki University School of Medicine (#M14005). 

Fourteen male WT and RG aged eight weeks were randomly divided into two groups. The ND group was fed a regular chow diet with 10% total calories from fat, while the other group was fed a HFD (Research Diets, Inc., New Brunswick, NJ, USA) with 60% of calories from fat for eight weeks to generate the DIO mouse model. The pancreas was resected, and blood was drawn from the right gastro-omental artery. Plasma and pancreatic tissues were stored at −80 °C

### 4.3. Measurement of Blood Glucose, Insulin, Total Cholesterol, and Triglycerides

Blood glucose, plasma TG, TC and insulin were measured at the end of the experimental period with previously described methods [50]. TG and TC were measured with an automated analyser for clinical chemistry (SPOTCHEM EZ SP−4430, ARKRAY Inc., Kyoto, Japan). The 2 g/kg oral glucose tolerance test and 1 U insulin tolerance test were conducted at the very start (week 1) and end (week 8) of the experiment [51]. 

### 4.4. Isolation and Culture of Mouse PSCs

PSCs from mice were isolated using a modification of the method described by Apte et al. [51]. Briefly, pancreatic tissue from mice and rats was minced and digested with 0.02% Pronase, 0.05% Collagenase P and 0.1% DNAse for 50 min. The cell suspension was layered with a 9-mL 28.7% (wt/vol) solution of Nycodenz (Axis-Shield, Oslo, Norway) and centrifuged at 1400× *g* for 20 min. Separated PSCs were washed and resuspended in DMEM/F12 containing 10% FBS, 4 mmol/L glutamine, and antibiotics (penicillin 100 U/mL and streptomycin 100 µg/mL). PSCs were cultured with 4 × 10^4^/cm^2^ maintained at 37 °C in a humidified atmosphere of 5% CO_2_/95% air. The purity of the isolated PSCs was determined by staining for αSMA and lipid droplets with oil red O staining. Only isolations with purity >95% were used for further experiments.

### 4.5. Activation of mPSCs and Cytokine Secretion 

Total RNA was extracted from isolated PSCs from DIO mice and controls or PSCs stimulated with AGE at the indicated concentration (#2221−10, BIoVision, Milpitas, CA, USA) after 80% confluence with TRIzol (Thermo Fisher Scientific, Waltham, MA, USA). Subsequently, cDNA was generated with the SuperScript^®^ VILO cDNA Synthesis Kit (Thermo Fisher Scientific). Quantitative PCR (qPCR) was performed with a previously described method [52,53]. Commercially available probe sets of mice (Gene expression assays, Thermo Fisher Scientific) for TGF−β, αSMA, collagen Type 1A1, RAGE, IL−6 and an internal standard of β2 microglobulin were mixed with cDNA Thunderbird Probe qPCR Mix (Toyobo Co. Ltd., Osaka, Japan). For cytokine secretion from PSCs, after 8 h of starvation with 0.5% FCS, PSCs were stimulated with 50 µg/mL AGEs for 6 h. The supernatant was filtered through 0.22 μm filters and stored at −80 °C. The concentrations of TGF−β and IL−6 were evaluated by ELISA following the manufacturer’s protocol (SEA124Hu ELISA kit for Transforming Growth Factor Beta1, Claud−Clone Crop., Katy, TX, USA and IL−6 mouse ELISA Kit Quantikine M 2nd Generation, R&D system, Minneapolis, MN, USA).

### 4.6. Coculture Experiments with mPSCs and Human PDAC Cell Lines

PSCs isolated from WT and RAGE−fed normal diets were grown in a dish until 80% confluence and stimulated with 50 μg/mL AGEs (BIoVision) or 50 μg/mL BSA for 12 h; subsequently, they were cultured for 24 h without stimulation, and the culture medium was harvested. The supernatant was filtered through 0.22 μm filters and stored at −80 °C as CM until use. The human PDAC cell lines BxPC−3 and AsPC−1 were obtained from the American Type Culture Collection (ATCC, Manassas, VA, USA). PDAC cell lines were cultured with AGE−CM or nAGE−CM for 24 h. PDAC cells were harvested for the quantitative EMT−related evaluation of mRNA and immunostained with anti−cytokeratin AE1/AE3 antibody (Agilent Technologies Inc., Santa Clara, CA, USA) and AlexaFluor 484-conjugated secondary antibody (Thermo Fischer Scientific). qPCR was performed with commercially available human probe sets for CDH1 and vimentin and an internal standard of β2 microglobulin (Thermo Fisher Scientific). 

### 4.7. Human PDAC Subjects 

We recruited 155 patients with PDAC who underwent pancreas resections between 2007 and 2017 and were available for pathological studies from the archive files of Hirosaki University Hospital. Eighty-three subjects were separated by nDM, 18 subjects had a history of diabetes of three years or less (sDM) and 54 subjects had a history of diabetes of more than three years (lnDM). Diabetic patients fulfilled the criteria of diabetes proposed by the Japan Diabetes Society [54].

### 4.8. Histopathological Assessment

The pathological diagnosis of PDAC and PanIN was re-evaluated according to the 2019 WHO classification of tumours of the digestive system and graded based on the UICC TNM classification of malignant tumours (8th edition) by three pathologists (C.U., H.M., and K.K.) [55]. The histological grade was divided into the three categories of well differentiated carcinoma (wel), moderately differentiated adenocarcinoma (mod), and por, based on the degree of tubular formation, mucin production and mitoses. The highest grade in the sections represented the histological grade of the individuals regardless of the proportion. PanIN was divided into three grades based on cytoarchitectural atypia. Venous invasion was assessed in the tumour sections stained with Elastica-von Gieson. Lymphatic invasion was evaluated on the immunostained sections for podoplanin (Leica Biosystems Inc., Wetzlar, Germany). The degree of invasion to venules and lymph vessels was graded as 0 (none), 1 (0–3 sites), 2 (3–6 sites) or 3 (6 sites<) using a 10× high-power field.

### 4.9. Immunohistochemical Analysis

An automated immunohistochemistry instrument was applied for immunohistochemical analysis (Bond autostainer, Leica Biosystems Inc.). The antibodies used in this study are listed in Supplementary Table S1. Sections were observed with an Axio−imager A-2 microscope (Carl Zeiss AG., Jena, Germany), and images were captured in 10 randomly selected frames of a 40× power field. The number of cells showing clearly positive cytoplasmic and cell membrane staining for vimentin and RAGE was counted and recorded as the number of positive cells per tumour or stromal cells. The positive reaction of E-cadherin was determined by Saito et al. (Score 0: no staining, 1+: weak and incomplete membrane staining in less than 10% of the invasive tumour cells, Score 2+: weak to moderate and complete staining of the membrane in more than 10% or strong complete homogenous membrane staining in no more than 10% of invasive cancer cells, Score 3+: strong complete homogenous membrane staining in more than 10% of the invasive tumour cells) [5]. A score of less than 2+ was judged as low expression of E−cadherin. Absolute lack of positive reaction in the whole section, including surrounding the adjacent parenchyma, was judged as “no expression”. 

### 4.10. PSC Activation Surrounding PanIN

Activation of PSCs surrounding PanIN (intermediate grade) was evaluated according to the degrees of αSMA−positive stromal cells surrounding PanIN based on the methods of Zhang et al. [7,56]. Seventy-six, 41 and 142 PanIN lesions were observed in 23 cases of nDM, 10 cases of sDM and 30 cases of lnDM, respectively. To exclude the influence of the diameter of the duct with PanIN, the formula described below was applied:

PSC activation score = [maximum width of αSMA positive area surrounding PanIN (μm)/major axis of duct (μm) × minor axis of duct (μm)] × 10^4^.

### 4.11. Statistical Analysis

Data are presented as the mean ± SD. All statistical analyses were conducted using JMP software (version 10.0.2, SAS Institute Inc., Cary, NC, USA). Continuous variables were compared with Student’s *t* test or the Mann–Whitney *U* test. Categorical variables were compared by chi-square analysis or the Mann–Whitney *U* test, where appropriate. Comparisons of average values between the two groups were analysed by the nonparametric Mann–Whitney *U* test. For multiple comparisons, two-way ANOVA with Bonferroni adjustment was used. A simple regression was performed for the correlation analysis. To evaluate the implication of metabolic syndrome markers in PSC activation in PDAC subjects, we adopted hierarchical “cluster analysis,” which enables grouping (clustering) of subjects with similar (but not identical) BMI, HbA1c, TC and TG values based on Ward’s method [57,58]. DFS was defined as the time elapsed between surgical resection and tumour recurrence. OS was calculated as the time between surgery and death from any cause. Survival curves were calculated using Kaplan–Meier analysis, and *p* values were determined by the log rank test for censored survival data. Multivariate survival analysis was performed by the Cox proportional hazard model. All tests were two-tailed, and a *p* value of < 0.05 was considered to be statistically significant.

## Figures and Tables

**Table 1:** Clinical profiles of DIO WT and RG.

	WT	WTHFD	RG	RGHFD
Body weight (g):				
Start (0 week)	21.3 ± 0.9 (*n* = 8)	21.2± 0.7 (*n* = 8)	21.4 ± 1.5 (*n* = 7)	21.3 ± 0.8 (*n* = 8)
End (8 weeks)	24.7 ± 1.4 (*n* = 7)	30.6 ± 2.8 * (*n* = 8)	25.8 ± 3.8 (*n* = 7)	30.3 ± 1.5 ^†^ (*n* = 8)
Fasting blood glucose (mmol/L) (8 weeks)	4.8 ± 0.5 (*n* = 4)	5.7 ± 1.0 * (*n* = 4)	4.4 ± 0.6 (*n* = 4)	5.7 ± 0.6 ^†^ (*n* = 4)
Fed blood glucose (mmol/L) (8 weeks)	10.7 ± 0.8 (*n* = 4)	12.9 ± 1.6 ^‡^ (*n* = 4)	10.9 ± 0.7 (*n* = 7)	13.5 ± 2.2 ^†^ (*n* = 8)
Triglycerides (mmol/L)(8 weeks)	1.4 ± 0.1 (*n* = 7)	3.1 ± 0.3 * (*n* = 8)	1.4 ± 0.1 (*n* = 7)	3.0 ± 0.2 ^†^ (*n* = 8)
Total cholesterol (mmol/L)(8 weeks)	0.3 ± 0.1 (*n* = 7)	0.5 ± 0.1 * (*n* = 8)	0.3 ± 0.1 (*n* = 7)	0.5 ± 0.1 ^†^ (*n* = 8)
Plasma insulin (ng/mL)(8 weeks)	1.8 ± 0.9 (*n* = 7)	2.9 ± 1.4 ^‡^ (*n* = 7)	2.0 ± 1.1 (*n* = 7)	2.7 ± 1.7 ^§^ (*n* = 7)

DIO: High fat diet induced obesity, WT: Wild type, WTHFD: High fat diet−fed WT, RG: RAGE−null mice, RGHFD: High fat diet−fed RG. Values were presented as mean ± SD. * *p* < 0.01 vs. WT, ^†^ *p* < 0.01 vs. RG, ^‡^ *p* < 0.05 vs. WT, ^§^ *p* < 0.05 vs. RG.

**Table 2:** Clinicopathological profiles of the subjects.

	nDM	sDM	lnDM
Number (Male/Female)	83 (34/49)	18 (10/8)	54 (27/27)
Age (years)	66.3 ± 8.1	66.6 ± 8.9	68.6 ± 8.2
Body mass index	22.5 ± 3.7	21.9 ± 3.0	22.7 ± 3.4
Diabetes duration (years)		1.8 ± 0.9	11.3 ± 7.0 *
HbA1c (NGSP, %)	5.7 ± 0.6	7.7 ± 1.5 ^†^	7.9 ± 1.6 ^†^
Triglycerides (mmol/L)	1.7 ± 1.0	1.3 ± 0.5	1.5± 1.0
Total cholesterol (mmoL/L)	5.2 ± 1.5	4.9 ± 1.5	4.6 ± 1.2 ^‡^
Tumor size (mm)	37.9 ± 16.7	39.4 ± 16.5	36.9 ± 15.5
ly−factor	2.1 ± 0.8	2.1 ± 0.8	2.3 ± 0.6
v−factor	2.0 ± 0.9	1.9 ± 0.9	2.4 ± 0.7 *^†^
Histological grade:			
wel−mod	81.9% (68/83)	88.9% (16/2)	44.4% (23/54)
por	18.1% (15/83)	11.1% (2/16)	55.6% (30/54) *^†^
T stage (UICC 8th):			
1/2	67.5% (56/83)	66.7% (12/18)	50.0% (26/32)
3/4	32.5% (27/83)	33.3% (6/18)	50.0% (26/54) ^‡§^
N stage:			
0	28.9% (24/83)	38.9% (7/18)	33.3% (18/54)
1/2	71.1% (59/83)	61.1% (11/18)	66.7% (36/54)

nDM: non−diabetic subjects, sDM: Short−term type 2 diabetic subjects, lnDM: Long−term type 2 diabetic subjects, NGSP: National Glycohemoglobin Standardization Program, wel: Well differentiated adenocarcinoma, mod: Moderately differentiated adenocarcinoma, por: Poorly differentiated adenocarcinoma. Values are presented as mean ± SD. * *p* < 0.01 vs. sDM, ^†^ *p* < 0.01 vs. nDM, ^‡^ *p* < 0.05 vs. nDM, ^§^ *p* < 0.05 vs. sDM.

**Table 3:** Clinical profiles of T2D subjects divided by cluster analysis.

	DMMS−	DMMS+	*p* Value
Number (male/female)	31(15/16)	23 (12/11)	
Age (years)	69.3 ± 8.0	68.4 ± 8.5	0.714
Body mass index	21.1 ± 2.8	25.1 ± 2.8	<0.001
Diabetes duration (years)	11.4± 7.1	11.0 ± 7.1	0.949
HbA1c (NGSP, %)	8.3 ± 1.8	7.7 ± 0.9	0.138
Triglycerides (mmol/L)	1.0 ± 0.3	2.3 ± 1.2	<0.001
Total cholesterol (mmol/L)	4.1 ± 0.9	5.5 ± 1.1	<0.001
Tumor size (mm)	38.0 ± 18.2	35.4 ± 10.8	0.549
Histological grade:			
wel-mod	51.6% (16/31)	34.8% (8/23)	0.022
por	48.4% (15/31)	65.2% (15/23)	
T stage (UICC 8th):			
T1−T2	58.1% (18/31)	41.9% (13/23)	<0.01
T3−T4	39.1% (9/31)	60.9% (14/23)	
N stage:			
0	38.7% (12/31)	26.1% (6/23)	0.049
1/2	61.3% (19/31)	73.9% (17/23)	

T2D: Type 2 diabetes, DMMS−: Long−term T2D subjects without metabolic syndrome, DMMS+: Long-term T2D subjects with metabolic syndrome, NGSP: National Glycohemoglobin Standardization Program, wel: Well differentiated adenocarcinoma, mod: Moderate differentiated adenocarcinoma, por: Poorly differentiated adenocarcinoma. Values are presented as mean ± SD.

**Table 4:** Univariate analysis of metabolic-pathological factors and disease-specific survival after resection of PDAC (log-rank test).

Variables	Median DFS (Month)	*p* Value
BMI: <24 vs. ≧24	9.8 vs. 8.0	0.472
History of T2D: (−) vs. (+)	12.4 vs. 8.0	0.002
HbA1c (%): <7.0 vs. ≧7.0	12.3 vs. 7.7	0.008
DMMS−: (−) vs. (+)	9.8 vs. 11.2	0.402
DMMS+: (−) vs. (+)	12.2 vs. 7.4	<0.001
TG (mmol/L): <1.7 vs. ≧1.7	11.2 vs. 15.8	0.458
TC (mmol/L): <5.7 vs. ≧5.7	10.7 vs. 7.6	0.499
E−cadherin expression: high vs. low	12.2 vs. 6.7	<0.001
Vimentin expression: low vs. high	13.0 vs. 7.2	0.011

PDAC: Pancreatic ductal adenocarcinoma, BMI: Body mass index, T2D: Type 2 diabetes, HbA1c: Glycated hemoglobin A1c, DMMS–: Long−term T2D subjects without metabolic syndrome, DMMS+: Long−term T2D subjects complicated with metabolic syndrome, TG: Triglycerides, TC: Total cholesterol.

**Table 5:** Univariate analysis of metabolic-pathological factors and overall survival after resection of PDAC (log-rank test).

Variables	Median OS (Month)	*p* Value
BMI: <24 vs. ≧24	18.9 vs. 16.8	0.826
History of T2D: (−) vs. (+)	21.8 vs. 12.6	<0.001
HbA1c (%): <7.0 vs. ≧7.0	20.5 vs. 12.4	0.001
DMMS−: (−) vs. (+)	19.15 vs. 17.5	0.292
DMMS+: (−) vs. (+)	20.0 vs. 11.7	<0.001
TG: <1.7 vs. ≧1.7	19.5 vs. 12.5	0.234
TC: <5.7 vs. ≧5.7	19.4 vs. 11.7	0.068
E−cadherin expression: high vs. low	20.6 vs. 12.4	<0.001
Vimentin expression: low vs. high	20.0 vs. 11.2	<0.001

PDAC: Pancreatic ductal adenocarcinoma, BMI: Body mass index, T2D: Type 2 diabetes, HbA1c: Glycated hemoglobin A1c, DMMS−: Long-term T2D subjects without metabolic syndrome, DMMS+: Long-term T2D subjects complicated with metabolic syndrome, TG: Triglycerides, TC: Total cholesterol.

**Table 6:** Multivariate analysis of clinicopathological factors and DFS after resection of PDAC (Cox proportional hazards model).

Variables	Hazard Ratio	95% CI	*p* Value
History of T2D: (−) vs. (+)	1.149	0.573–2.146	0.681
HbA1c (%): <7.0 vs. ≧7.0	0.980	0.540–1.883	0.949
DMMS+	1.528	0.840–2.725	0.163
E−cadherin low	1.351	0.787–2.298	0.029
Vimentin high	1.721	1.060–2.742	0.273

DFS: Disease fee survival, PDAC: Pancreas ductal adenocarcinoma, CI: Confidence interval, T2D: Type 2 diabetes, HbA1c: Glycated hemoglobin A1c, DMMS+: Long−term T2D subjects complicated with metabolic syndrome.

**Table 7:** Multivariate analysis of clinicopathological factors and OS after resection of PDAC (Cox proportional hazards model).

Variables	Hazard Ratio	95% CI	*p* Value
History of T2D: (−) vs. (+)	1.008	0.497–1.898	0.979
HbA1c (%): <7.0 vs. ≧7.0	1.337	0.689–2.733	0.398
DMMS+	1.609	0.900–2.839	0.107
E−cadherin low	1.184	1.246–3.089	0.004
Vimentin high	1.984	0.677–2.059	0.552

OS: Overall survival, PDAC: Pancreas ductal adenocarcinoma, T2D: Type 2 diabetes, CI: Confidence interval, HbA1c: Glycated hemoglobin A1c, DMMS+: Long−term T2D subjects complicated with metabolic syndrome.

## Data Availability

Not applicable.

## Supplementary Materials

The following are available online at https://www.mdpi.com/article/10.3390/ijms222111716/s1.

## Abbreviations

PDAC: Pancreatic ductal adenocarcinoma; T2D, Type 2 diabetes; PSCs, Pancreatic stellate cells; CAFs, Cancer associated fibroblasts; MFCs, Myofibroblast−like cells; αSMA, Alpha smooth muscle actin; RAGE, Receptor for advanced glycation end products; AGEs, Advanced glycation end products; HSCs, Hepatic stellate cells; EMT, Epithelial−mesenchymal transition; RG, RAGE−null mice; WT, Wild type littermate mice; ND, Normal diet; HFD, High fat diet; DIO, HFD−induced obese; TC, Total cholesterol; TG, Triglycerides; OGTT, Oral glucose tolerance test; ITT, Insulin tolerance test; CM, Conditioned media; nDM, Non−diabetic subjects; sDM, Short−term diabetic subjects; lnDM, Long−term diabetic subjects; wel, Well differentiated carcinoma; mod, Moderately differentiated adenocarcinoma; por, Poorly differentiated adenocarcinoma; DMMS−, lnDM without metabolic syndrome; DMMS+, lnDM complicated with metabolic syndrome; PanIN, Pancreatic intraepithelial neoplasia; DFS, Disease−free survival; OS, Overall survival.
