# Supplementary material for: Diabetes in Humans Activates Pancreatic Stellate Cells via RAGE in Pancreatic Ductal Adenocarcinoma
Source: Int J Mol Sci. 2021 Oct 28;22(21):11716. doi: 10.3390/ijms222111716 (PMC8584151; doi:10.3390/ijms222111716)
Supplement: Supplementary file 1 [file ijms-22-11716-s001.zip › ijms-1349274-supplementary.pdf]

Supplementary Table S1. Antibody List

Antibody name	Species	Catalog No.	Vendor	Experiments	Dilutions
CK AE1/AE3	Mouse	IR053	Agilent Technologies (CA, USA)	IF	1:200
Podplanin (D2-40)	Mouse	413451	Nichirei Corp. (Tokyo, Japan)	IHC	1:4
◎SMA	Mouse	M0851	Agilent Technologies (CA, USA)	IHC	1:500
E-cadherin	Mouse	M3612	Agilent Technologies (CA, USA)	IHC	1:250
Vimentin	Mouse	NCL-L-VIM-V9	Leica Biosystems (IL, USA)	IHC	1:200
RAGE	Rabbit	PA1069 AGER	Boster Biological Technology Ltd. (CA, USA)	IHC	1:500
Alexa Fluor 488 anti-mouse IgG (H+L)	Donkey	A21202	Thermo Fisher Scientific (MA, USA)	IF	1:1000

CK, Cytokeratin; ◎SMA, Alpha smooth muscle actin; RAGE, Receptor for advanced glycated end products; IF, Immunofluorescence; IHC, Immunohistochemistry.

Supplementary Figure S1

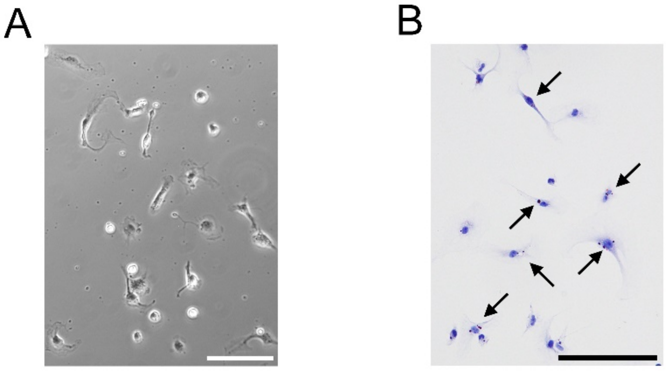

The morphology of PSCs was spindle and ameboid (**A**). Those cells contained lipid droplet which was positive for oil red-O staining (arrows) (**B**). The scale bar represents 100 μm.
